# Neurophysiological Measures of the Perception of Antismoking Public Service Announcements Among Young Population

**DOI:** 10.3389/fnhum.2018.00231

**Published:** 2018-08-29

**Authors:** Giulia Cartocci, Enrica Modica, Dario Rossi, Patrizia Cherubino, Anton Giulio Maglione, Alfredo Colosimo, Arianna Trettel, Marco Mancini, Fabio Babiloni

**Affiliations:** ^1^Department of Molecular Medicine, Sapienza University of Rome, Rome, Italy; ^2^Department of Anatomical, Histological, Forensic & Orthopedic Sciences, Sapienza University of Rome, Rome, Italy; ^3^BrainSigns Srl, Rome, Italy; ^4^Department of Computer Science, Hangzhou Dianzi University, Xiasha Higher Education Zone, Hangzhou, China

**Keywords:** EEG, alpha, heart rate, galvanic skin response, emotion, eye tracking, cognitive effort, approach/avoidance

## Abstract

Tobacco constitutes a global emergency with totally preventable millions of deaths per year and smoking-related illnesses. Public service announcements (PSAs) are the main tool against smoking and by now their efficacy is still assessed through questionnaires and metrics, only months after their circulation. The present study focused on the young population, because at higher risk of developing tobacco addiction, investigating the reaction to the vision of Effective, Ineffective and Awarded antismoking PSAs through: electroencephalography (EEG), autonomic activity variation (Galvanic skin response—GSR- and Heart Rate—HR-) and Eye-Tracking (ET). The employed indices were: the EEG frontal alpha band asymmetry and the frontal theta; the Emotional Index (EI), deriving from the GSR and HR signals matching; the ET Visual Attention (VA) index, based on the ratio between the total time spent fixating an area of interest (AOI) and its area. Smokers expressed higher frontal alpha asymmetry values in comparison to non-smokers. Concerning frontal theta, Awarded PSAs reported the highest values in comparison to both Effective and Ineffective PSAs. EI results highlighted that lowest values were expressed by Heavy Smokers (HS), and Effective PSAs obtained the highest EI values. Finally, concerning the Effective PSAs, regression analysis highlighted a correlation between the number of cigarettes smoked by participants (independent variable) and frontal alpha asymmetry, frontal theta and EI values. ET results suggested that for the Ineffective PSAs the main focus were texts, while for the Effective and Awarded PSAs were the visual elements. Results support the use of methods aimed at assessing the physiological reaction for the evaluation of PSAs images, in particular when considering the smoking habits of target populations.

## Introduction

Tobacco use spread is becoming a global emergency, as witnessed by data provided by the World Health Organization (WHO) describing it as the main cause of preventable death at a global level. In fact, each year worldwide there are around 6 million deaths and hundreds of billions of dollars of economic damage related to smoking^1^ (WHO report 2011). But the good news is that the millions of deaths per year and smoking-related illnesses are totally preventable. Neuroscience is recently joining this battle beside more traditional fields, such as medicine and psychology. In particular, the approach used in the present article investigated the study of a multiple-parameters physiological reaction to antismoking public service announcements (PSAs) (Hornik, 2002). Such PSAs have been broadcasted since the 1950s, but results in terms of effectiveness, have been extremely variable, ranging from highly effective to ineffective campaigns, resulting in the second case in a waste of public money and time. Encouragingly, research has shown that antismoking advertising can be successful both in adult (Bala et al., 2013) and young people (Brinn et al., 2010). Additionally, WHO data^1^ report that effective anti-tobacco mass media campaigns are spread among almost 28% of the world’s population. Traditionally, effectiveness is measured only months (if not years) after PSAs circulation, through classical methods: questionnaires and several antismoke-related metrics, concerning health issues as well as economic improvement in the smoking-related health burden. Cerebral measurements can be a “predictor” of the success of an audiovisual material in a particular population, as recently shown (Berns and Moore, 2012). In particular, those researchers recorded a group of adolescents by functional Magnetic Resonance Imaging (fMRI), evidencing a significant correlation between the activity within the ventral striatum during the listening to unknown songs, and the number of music albums sold in the following 3 years (Berns and Moore, 2012).

As mentioned above, methods of PSAs evaluation are often performed *a posteriori*, whilst an appropriate pre-testing of the PSA material would be extremely useful to check the impact of the particular creative solutions toward the target populations.

It could be of interest to understand if the PSA assessment (e.g., effective, ineffective) can be performed through the study of the neurophysiological reaction to the exposure to the PSA itself. It could be hypothesized that possible different cerebral patterns could be obtained in response to different kind of effective (e.g., successful) or ineffective PSAs by using neurophysiological measurements, such as electroencephalogram (EEG).

Therefore, the obtainment of measurable neurophysiological parameters, collected through a direct analysis of the measured cerebral/emotional/Visual Attention (VA) in response to the observation of PSAs, represents a matter worthy of study. Through such neuroscience techniques several aspects related to the commerce can be investigated: target population’s gender (Vecchiato et al., 2014a; Cartocci et al., 2016a), culture (Han and Shavitt, 1994; Vecchiato et al., 2011a) and age (Cherubino et al., 2016a); fragments of interest (Vecchiato et al., 2010, 2011b, 2014a); the brand (Paulus and Frank, 2003); the price (Reimann et al., 2011); scenes targeting and speaker’s gender (Cherubino et al., 2016b); purchasing attitudes of the subjects (Knutson et al., 2007) and pre-retail testing (Baldo et al., 2015). The capability of EEG techniques to detect different patterns between smokers and non-smokers have been already provided from event related potentials (ERP) studies, in which the amplitude of the P300 resulted lower in smokers than in non-smokers (Anokhin et al., 2000; Jang et al., 2007; Guney et al., 2009). Importantly, this difference can be affected by the stimulus category, as evidenced by an ERP study in which it has been shown a signicant smoking cue-reactivity of the P412, a P300-like wave correlation with unpleasantness-pleasantness in reaction to the cue (Warren and McDonough, 1999). In the present study, two EEG indices have been employed for the evaluation of the response to the antismoking PSAs vision: the frontal alpha asymmetry and the frontal theta activity. The frontal alpha asymmetry index definition derives from Davidson et al.’s (1990) studies, showing that frontal asymmetry activity between the two hemispheres implies a different motivational tendency toward the proposed stimulus. Behind this statement there are evidences reported by several authors (for a review Davidson and Irwin, 1999; Coan and Allen, 2004; Smith et al., 2017) of the existence of two distinct neural systems, localized in the left and right hemispheres, responsible for an approach-related and a withdrawal-related motivation respectively. In accordance with these observations, the prefrontal cortex (PFC) plays a pivotal role in the circuitry that mediates both positive and negative motivation. In particular, various studies evidenced a relative increase in the left PFC activation in correspondence of the positive motivation, while an augmented right-sided anterior activation was observed during the negative motivation (Davidson et al., 1990; Davidson, 1995, 1998), and regardless of the positive or negative dispositional affect (Sutton and Davidson, 1997). Concerning this model, it has been repeatedly studied on different Participant samples and experimental conditions (Davidson, 1992; Harmon-Jones and Allen, 1998; Harmon-Jones and Sigelman, 2001; Harmon-Jones et al., 2003; Cacioppo, 2004; Hagemann, 2004; Harmon-Jones, 2004; Harmon-Jones et al., 2010; Reznik and Allen, 2017), both concerning state and trait issues, depending on the experimental set-up. For instance, in reaction to the vision of positive and negative emotional short films, it has been found a higher right-sided frontal and anterior temporal region activation (and therefore a motivation of withdrawal) in response to disgust elicited among participants, in comparison to happiness. Conversely, happiness produced higher left-sided activation (and therefore a motivation of approach) in comparison to disgust (Davidson et al., 1990). The frontal alpha asymmetry investigation has been also applied to the study of personality traits in children, finding evidence that the extent of the prefrontal alpha asymmetry was predictive of the attitude to undertake play activities with novel toys (Davidson, 1995). In addition, it has been evaluated the tendency to the approach motivation by modulating the frontal alpha asymmetry under three experimental conditions characterized by a low-, a neutral and a high-approach positive mindset (Harmon-Jones et al., 2008). Results from the just mentioned study pointed out that the high-approach positive mindset produced a greater relative left frontal activity in comparison to the other conditions. The application to advertising material of the frontal alpha asymmetry index, have been already repeatedly reported (Vecchiato et al., 2010, 2011a, 2012, 2014a; Silberstein and Nield, 2012; Cartocci et al., 2016c). To recapitulate, frontal alpha asymmetry indicating higher left activity would return frontal alpha asymmetry index positive values, suggesting a positive motivation toward the stimuli, *vice versa* higher right activity would be reflected by negative frontal alpha asymmetry values, suggesting a negative motivation in response to the stimuli.

The second EEG index considered in this study is the amount of EEG power in the theta band collected from the prefrontal scalp areas, hereafter named for brevity as “frontal theta.” Higher values of frontal theta have been connected to higher levels of task difficulty (Klimesch, 1999) and itrepresents a marker of cognitive processings occurring in correspondence of mental fatigue increase during a visual task (Wascher et al., 2014). The increase in the frontal theta in parallel of the mental effort increase has been specifically identified as peculiar of the mental effort, since the physical effort produces instead an increase in alpha and beta one power (Smit et al., 2005). Furthermore, it has been seen frontal theta to rise in correspondence to the task complexity (Gevins et al., 1998; Aricò et al., 2016; Toppi et al., 2016; Vecchiato et al., 2016). Additionally, Gevins and colleagues provided evidences of frontal theta activity increase in correspondence of high task load in an *n*-back task, further supporting the relation between this index and the mental workload concept (Gevins et al., 1997, 1998; Smith and Gevins, 2005a,b). Moreover, during a dichotic listening task aimed at the workload manipulation, it has been shown an increase of the theta power in medium and high workload conditions in comparison to a screening condition (Käthner et al., 2014). The frontal theta as index of effort and processing has been already applied to a series of studies in different field of research. For instance, in the neuroaesthetics in response to auditory literature stimuli (Cartocci et al., 2016d); in avionic and car driving for the detection of the effort employed in the execution of flight simulation and driving tasks (Borghini et al., 2013, 2014, 2017; Aricò et al., 2014; Di Flumeri et al., 2015; Sciaraffa et al., 2017); in different challenging listening conditions both in normal hearing than in hearing impaired participants (Cartocci et al., 2015; Wisniewski et al., 2015, 2017) and to human–computer interaction (Gevins and Smith, 2003) studies.

It is well known from empirical observations in various fields of application, that the emotional involvement boosts the memorization of audiovisual stimuli. Also in consumer neuroscience, positive or negative emotional processing during the watching of commercial advertisements is crucial for the development of stable memories (Kato et al., 2009). Furthermore, very often in response to advertising unconscious reactions take place (Bargh and Chartrand, 1999). In fact, an “unconscious emotion” occurs when there is a decoupling between brain systems mediating unconscious “liking” (for instance, the nucleus accumbens and its connections) and conscious awareness (Berridge and Winkielman, 2003). The link between autonomic nervous system activity and emotional states has been suggested since the end of the XIX century by the psychologist James (1884). Discrete and dimensional theoretical models of emotions have been designed, implying for emotional states respectively: peculiar physiological, behavioral and experiential correlates for the discrete models; while a combination of affective dimensions, mainly valence and arousal, for the dimensional models (Posner et al., 2005; Mauss and Robinson, 2009; Hamann, 2012). Typically, electrodermal (corresponding to the activity of the sweat glands) and cardiovascular responses are at the basis of the mostly used indices of activation of the autonomic nervous system. The electrodermal activity is often measured by the Galvanic Skin Response (GSR), and it represents an index of changes in sympathetic arousal (Lang et al., 1993; Bradley and Lang, 2000; Bradley et al., 2001; Critchley, 2002; Frazier et al., 2004; Gomez et al., 2005), indipendently of the valence of the emotional stimulus. In addition, the heart rate (HR) has been evidenced as an index of sympathetic and parasympathetic activity (Mauss and Robinson, 2009), and the correlation between the HR and the emotional valence of a stimulus has been repeatedly shown (Palomba et al., 1997, 2000; Baldaro et al., 2001; Frazier et al., 2004; Gomez et al., 2005; Britton et al., 2006; Kreibig et al., 2007). Starting from this assumption, in the present research has been adopted an autonomic index, resulting from the matching of the GSR and the HR. These two signals reflect the emotional response to stimuli (see for a review Mauss and Robinson, 2009) and the resulting Emotional index (EI), has been conceived starting from Russell and Barrett’s circumplex model of affect (Russell and Barrett, 1999). In this model the HR is plotted on the *x*-axis relative to the emotional valence in the Russel’s plane, while the GSR on the *y*-axis, reflecting information concerning the stimuli arousal (low or high activation; Astolfi et al., 2008; Vecchiato et al., 2013, 2014c). The EI has been already applied for instance to the testing of auditory literary stimuli (Cartocci et al., 2016d) and to TV commercials (Vecchiato et al., 2014a,b; Cherubino et al., 2016a,b). The adoption of consumer neuroscience techniques, in order to study neurophysiological features of effectiveness in antismoking PSAs, has been already approached by the employment of the frontal alpha asymmetry, frontal theta and EI indices in a reduced sample of participants and spots, supporting the potentiality of this method for the PSAs evaluation (Cartocci et al., 2016b,c).

Furthermore, by Eye-Tracking (ET) investigation it is possible to directly measure attention, that is a substantial precursor to information processing, memory and other decisive activities in regulatory science (Klein et al., 2015; Meernik et al., 2016). The saliency of areas containing health-warning cues in PSAs appears as a fundamental feature to be investigated since it is preliminary to understand, recall and use of the information for making health-related decisions (Kees et al., 2010). Previous studies using ET on printed tobacco advertisement in a sample of adolescents showed that less than 10% of the total advertisement viewing time was spent on the warning, and that 44% of the participants did not watch the warning at all (Fischer et al., 1989). Another study on adolescents showed the influence of the novelty in terms of font and colors manipulation on the percentages of fixation (Krugman et al., 1994). Additionally, the manipulation of graphic warnings rather than only the textual ones, resulted in higher fixation numbers toward the graphic warnings compared to the texts (Peterson et al., 2010). Therefore, aim of this study was to investigate the physiological (EEG, autonomic and ET) reaction to the exposure to selected antismoking PSAs in a young sample composed by high school and university students, in order to investigate eventual patterns typical of effective or ineffective antismoking PSAs.

In this study, it has been focused on the young population, because presenting the higher risk of developing tobacco addiction^2^. Moreover, adolescents are particularly vulnerable since living a transitional phase that could lead them to believe that the use of tobacco products can provide satisfaction of their social and psychological requirements (e.g., peer acceptance, popularity and positive self-image)^2^,^3^,^4^. Furthermore, with respect to the epidemiology of the country in which the study took place (Italy), the majority of the Italian smoking beginners range between 15 years and 17 years old, with no difference between genders^5^ (DOXA 2015).

We hypothesize that PSAs characterized by a different degree of effectiveness (as classified on the basis of independent data already available from independent sources), would produce different neurophysiological patterns in the EEG, autonomic and ET results in groups characterized by different smoking habit. In particular we make the following hypothesis:

1. Hypothesis 1: the theory of psychological reactance (Brehm and Brehm, 2013) predicts that messages felt as reducing or threatening personal freedoms (such as, the choice to smoke) produce reactance, a motivational state prompting individuals toward re-establishing the lost or threatened freedom. In accord to this theory, we hypothesize that Heavy Smokers (HS) would present a more negative emotional reaction to antismoking PSAs in comparison to light and No Smokers (NS);
2. Hypothesis 2: previous studies showed the influence of the level of nicotine dependence on the EEG activity (Haarer and Polich, 2000), and the different neuronal activity in response to smoking-related cue in highly dependent smokers, moderately dependent smokers and NS (Vollstädt-Klein et al., 2011). Therefore, we hypothesize that HS would show an increased approach tendency (higher frontal alpha asymmetry values) toward smoking-related contents in comparison to both light and NS;
3. Hypothesis 3: Wang et al. (2013) showed a correlation between dorso-medial PFC activity in correspondence of the exposure to anti-smoking messages and decreased levels of urinary cotinine at a 1-month follow-up, so supporting the possibility to identify effective anti-smoking communications *a priori*. Since the involvement of frontal theta in stimuli processing (Wascher et al., 2014), we hypothesize that Effective PSAs would show increased frontal theta activity in comparison to Ineffective PSAs, mirroring the occurrence of the anti-smoking message processing.

## Materials and Methods

### Participants

The experimental sample was composed by 39 volunteers (12 M, 27 F; average age = 18.308 ± 2.726 years old, min = 15, max = 24 years old), 13 HS, 11 Light Smokers (LS), 15 NS. Participants smoking more than five cigarettes per day were classified as HS; participants who did not smoke any cigarette were classified as NS. Starting from these classification, the HS group smoked an average of 9.703 ± 3.728 and the LS group an average of 1.127 ± 1.456 cigarettes/day. All subjects were given of detailed information about the study and signed an informed consent. The experiment was performed in accord to the principles outlined in the Declaration of Helsinki of 1975, as revised in 2000, and it was approved by the Sapienza University of Rome ethical committee in charge for the Department of Molecular Medicine. During the execution of the test, participants were sitting on a comfortable chair in front of a computer screen and they were not instructed with any particular task, just to be relaxed and to restrict head and body movements as much as possible.

### Protocol

Participants were asked to watch a video composed by six neutral images taken from IAPS database (Lang et al., 2008) used as baseline, followed by a train of 10 antismoking PSAs images displayed in a randomized order (so to prevent in the participants reaction the eventual bias attributable to a positional effect), followed by the images baseline. Images were displayed for 9 s each and between each pair of them a black cross on a white field was shown, so to reestablish a central fixation point. The stimuli were presented on a 19″ flat screen with a distance from the subject varying from 50–60 cm. Before the exposure to the experimental stimuli, participants were asked to look at a black screen for 60 s, and the EEG activity recorded in correspondence of this open eyes condition was then used for the IAF calculation (Goljahani et al., 2012).

For the EEG, autonomic responses and ET investigation six images have been selected, because presenting both text and visual elements. In particular, each image have been identified two kinds of areas of interest (AOIs): “visual” (such as the image of a lady who underwent a tracheotomy; or the picture of a young man with a sarcastic expression, depicted while smoking a cigarette) and “text” (text of the claim or the antismoking message). PSAs have been divided in three groups: “Effective,” “Ineffective” and “Awarded.” The “Effective” and “Ineffective” PSAs were the PSAs that produced the best and the worst documented answers by the general public, respectively, according to the published documentation available (Coffman, 2002; Varcoe, 2004). Such answers are measured through a number of metrics including the number of phone calls to the quit-smoking centers after the campaign, the decrement/increment of ambulatory visit for the attempt to quit smoking and so forth. The third kind of PSAs investigated has been previously classified as “Awarded” on the basis of the evaluation obtained by specialized committees, expressed as number of prizes received. In particular, the selected PSAs were:

-Effective:

- Centers for Disease Control and Prevention (CDC), Terry (USA 2012–2015, fear arousing appeal and narrative/experiential communication style): the image portraits a sick lady presenting the signs of a tracheotomy, flanked by the sentence “Don’t tell people smoking is bad, show them.”
- NTC, Kids are fast learners (Australia 1997, paternalistic communication style): the image displays a child holding a cigarette in his right hand and carefully looking at it; on the bottom of the picture there is the sentence “Kids are fast learners.”

-Ineffective:

- Feel free to say no (European Commission 2003, communication style aiming at the identification from young people with the represented young models): the picture is composed by three sections each depicting young people flanked by the icon of the campaign and the following antismoking sentences: “Who wants to be a looser?,” “We won’t let them spoil our fun!,” “Who wants to be addicted?.”
- Tobacco is wacko (USA 2000, paradoxical communication style): it is a drawing of a coughing boy surrounded by the sentence “Tobacco is wacko if you are a teen.”

- Awarded:

- Fatty Cigarette, British Heart Foundation (UK 2003, symbolic communication style): the picture represents a cigarette recalling an artery filled by fat, below there was the stament “Every cigarette we smoke makes fatty deposits stick in our arteries.”
- Quit the Denial, BBDO Toronto (Canada 2013, sarcastic communication style): the image depicts a young man smoking a cigarette with above the sentence “I’m not a smoker” and below him the phrase “Except every Thursday, Friday, Saturday night.”

At the end of the observation of the PSA images, it has been asked to the participants to spontaneously recall the PSAs writing a list of the images just seen.

### EEG Recordings and Signal Processing

The EEG activity was recorded using a 10-electrodes-based EEG frontal band (Fpz, Fp1, Fp2, AFz, AF3, AF4, AF5, AF6, AF7, AF8) by means of a portable 24-channels system (BEmicro, EBneuro, Italy). The signals have been acquired at a sampling rate of 256 Hz and the impedances were kept below 10 kΩ. First, a notch filter (50 Hz) was applied in order to reject the main current interference. Second, the gathered signal has been digitally band-pass filtered by a 5th order Butterworth filter ([2÷30] Hz), in order to reject the continuous component as well as high-frequencies interferences, such as muscular artifacts. Then, the Independent Component Analysis (ICA) has been applied to EEG data in order to identify and remove other artifacts-related components, such as blinks and eye movements, since their contribution is overlapped to the EEG bands of interest in the present study (Di Flumeri et al., 2016a). In order to take into account any subjective difference in terms of brain rhythms, for each subject the Individual Alpha Frequency (IAF) was computed on the 60-s-long Open Eyes segment (Goljahani et al., 2012), recorded at the beginning of the experimental task, in order to define the EEG bands of interest according to the method suggested in the current scientific literature, i.e., each band is defined as “IAF ± x,” where IAF is the Individual Alpha Frequency, in Hertz, and × an integer in the frequency domain (Klimesch, 1999). Thus, the EEG activity was divided, by filtering the EEG signals in the time-domain, in two main frequency bands: theta [IAF-6 ÷ IAF-2 Hz] and alpha [IAF-2 ÷ IAF+2 Hz].

To summarize the activity of the cortical areas of interest in a specific frequency band, the Global Field Power (GFP) was then computed. This is a measurement introduced by Lehmann and Michel (1990) some decades ago to summarize the synchronization level of the brain activity over the scalp surface. In general, the measure of the GFP corresponds to the spatial standard deviation, and it estimates the quantity of activity at each time point in the potential field, simultaneously considering data from all recording electrodes, producing a reference-independent descriptor of the field (Skrandies, 1990). The underlying idea of this procedure is that potential fields with few field lines presumably contain little information while scalp fields displaying much activity reflect the synchronous activation of a large number of intracranial neuronal elements. The GFP has been used in studies of perceptual, attentional, cognitive processing (e.g., Dierks and Maurer, 1989; Skrandies, 1991; Rodin, 1991; Michel et al., 1993; Rau et al., 2002; Boonstra et al., 2013; Ahonen et al., 2016) as well as in clinical studies (e.g., Rodin, 1990; Maurer et al., 1990; Strik et al., 1994; Favrod et al., 2017; Giroud et al., 2017; Iannilli et al., 2017). The GFP constitutes a good index for the temporal determination of information from cognitive studies, furthermore it has become a commonly used parameter for the time-domain analysis of EEG (as in the present study), since it allows to identify the maps of maximal electric field strength (hilliness).

In particular, in this study the GFP has been computed from a specific set of electrodes (the set depends on the investigated brain area, in the following it will be specified for each index) by performing the sum of squared values of EEG potential at each electrode, averaged for the number of involved electrodes, resulting in a time-varying waveform related to the increase or decrease of the global power in the analyzed EEG. The GFP formula is presented in the following:

$$GFP_{\vartheta ,\;Frontal}(t)=\frac{1}{N}\sum_{i=1}^{N}x_{i,\vartheta }{\left(t\right)}^{2}$$

where *ϑ* is the considered EEG band, *Frontal* is the considered cortical area, *N* is the number of electrodes included in the area of interest (AOI; in this example the Frontal area), and *i* is the electrodes’ index. Also, in the present study the GFP function, initially with the same time-resolution of the EEG gathered signal (i.e., equal to the sampling rate of 256 Hz), has been averaged on 1-s-long signal windows, in order to comply with the EEG signal stationarity hypothesis (Elul, 1969). According to the cognitive phenomenon to investigate, in the following the considered electrodes and brain rhythms are described.

It has been repeatedly evidenced in literature, thanks to the application to several kinds of stimuli (e.g., Davidson, 2004; Borghini et al., 2015; Maglione et al., 2015; Di Flumeri et al., 2016b, 2017), that the frontal cortex constitutes AOI for the analysis of the approach or withdrawal attitude (Davidson, 2004), while the frontal theta as index of cerebral effort and mental processing (Klimesch, 1999; Gevins and Smith, 2003; Wascher et al., 2014).

The formula defining the frontal alpha asymmetry index is the following:

frontal alpha asymmetry=GFPα_right−GFPα_left

where the GFPα_right and GFPalpha_left stand for the GFP calculated among right (Fp2, AF4, AF6, AF8) and left (Fp1, AF3, AF5, AF7) electrodes respectively, in the alpha (α) band. The frontal alpha asymmetry index was estimated for each second, and then standardized on the basis of the baseline (the IAPS images) EEG activity acquired at the beginning and at the end of the experiment. Higher frontal alpha asymmetry values, reported by the subjects, stood for an approach motivation toward the stimulus, while lower frontal alpha asymmetry values for a withdrawal motivation (Davidson, 2004).

To evaluate the mental effort/processing, EEG activity in the theta band over all the frontal electrodes (Fp2, AF4, AF6, AF8, AF7, AF3, Fp1, AF5) has been considered for the GFP computation. Thus, the obtained GFP values have been standardized according to the baseline EEG activity acquired at the beginning and at the end of the experiment, similarly to the procedure applied for the frontal alpha asymmetry index. An increase in the frontal theta (i.e., mental effort) would imply an increase in the task difficulty (Wisniewski et al., 2015).

### HR and GSR Recordings and Signal Processing

GSR and Electrocardiographic (ECG) signals were recorded with a sampling rate of 128 Hz through a NeXus-10 (Mindmedia, Netherlands) system. For the recording of the cardiac activity, disposable electrodes (Mindmedia company) were placed on the subject’s wrists. In order to estimate the HR from the ECG signal, it has been used the Pan-Tompkins algorithm (Pan and Tompkins, 1985). The constant voltage method (0.5 V) was employed for the acquisition of the GSR, also named skin conductance as the signal related to the skin sweating. The electrodes were placed to the palmar side of the middle phalanges of the second and third fingers, on the non-dominant hand of the participant, according to published procedures (Boucsein et al., 2012). Employing the LEDAlab software (Benedek and Kaernbach, 2010), the tonic component of the skin conductance (Skin Conductance Level, SCL) was estimated. The circumplex model of affect plane was adopted to collapse information about a stimulus deriving from SCL and HR (Russell and Barrett, 1999; Posner et al., 2005). In this model, the *x* axis reported the HR values, reflecting the valence dimension of the stimulus-related emotional state, while the *y* axis reported the SCL values, mirroring the related arousal dimension (for a review see Mauss and Robinson, 2009). Adopting this theoretical framework, it was possible to obtain a synthetic monodimensional variable, named the EI, providing information concerning the emotional status of a participant, as defined in previous studies (Vecchiato et al., 2014a). The EI has been calculated as defined in the just mentioned previous studies so to vary between −1 and +1. EI results interpretation predicts that higher values would mirror a more positive and engaging emotion experienced by the subject, while lower values would reflect more negative and less engaging emotion experienced by participants.

### Eye-Tracking Recordings and Data Processing

ET data have been acquired by a remote eye-tracker (Eye Tribe) with a sampling frequency of 30 Hz, in order to identify eye fixations on the proposed stimuli. As first step, all the artifactual or not physiological points of gaze were automatically removed. Then, all the points belonging eye movements (such as saccades or smooth pursuit) were removed by an Dispersion-Threshold Identification (I-DT) algorithm (Salvucci and Goldberg, 2000). Such algorithm uses two thresholds, a temporal one, set at 100 ms, and a spatial one, set at 60 pixels (for the identification of a fixation point on a picture (Rayner, 2009). Then, ET data were analyzed through a software for the extraction of information about fixations in each AOI, such as number of fixations and total time of fixation spent on each AOI. Each AOI (graphical and text) has been measured in relation to the size of the entire image in order to weight the time of fixation spent on the AOI. This since evidences show that the size of a visual target influences the amount of time spent on it (Lohse, 1997). Therefore, an index of VA has been defined as follows:

VA=%TFD=%AREA

where %Total Fixation Duration (TFD) is the total time spent fixating an AOI weighted on the total time the image is shown on the screen, and %AREA is the area of the AOI weighted on the total dimension of the image. This formula returns a non-dimensional index suitable for the comparison among different AOIs. It can be predicted that elements gaining higher VA values would have more visual saliency for the participants. In addition, since the essence of a scene is acquired very quickly (about 100 ms) in the first fixations (Pieters and Wedel, 2012), it was hypothesized that elements characterized by higher VA values would elicit more interest in the subjects, with a top-down mechanism.

### Statistical Analysis

ANOVA test has been performed on the frontal alpha asymmetry, frontal theta, EI and ET data. The between variable was the “Smoking Habit,” with three levels: HS, LS and NS; the within variable was the “PSA kind,” with three levels: Effective, Ineffective and Awarded. Concerning ET data also the within variable Element (with two levels: Text and Visual) has been included in the ANOVA analysis. Duncan *post hoc* has been employed on the statistically significant results from ANOVA. Linear regression analysis has been performed among the number of cigarettes smoked by participants (independent variable) and frontal alpha asymmetry, frontal theta and EI values (dependent variable), in order to investigate the possible correlation between these two kinds of data. Since the number of factors considered in the study is lower than 4, the ANOVA test has a sufficient statistical power to deal with the analysis also of moderately small number of participants (Zar, 2000). Finally, the sample size employed in the study has been set on the basis of the best practice adopted in numerous previous studies in literature, concerning the study of the electroencephalographic and autonomic indices employed in the research. In particular, in scientific literature for frontal alpha asymmetry, frontal theta as well as EI has been demonstrated as a sample size of 12–15 persons could be enough to capture less than 10% variation of these indexes (e.g., Gevins et al., 1997; Dussault et al., 2005; Domino et al., 2009; Maglione et al., 2014; Wascher et al., 2014; Cartocci et al., 2015, 2017; Wisniewski et al., 2015, 2017; Soria Morillo et al., 2016; Di Flumeri et al., 2016b; Cherubino et al., 2016a; Liu et al., 2017; Aghajani et al., 2017; Mennella et al., 2017).

## Results

### Frontal Alpha Asymmetry

**Figure 1:**
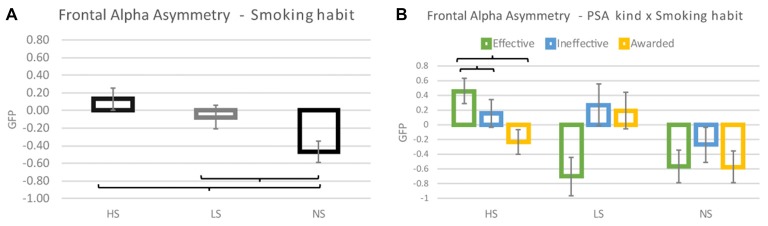
Frontal alpha asymmetry results. **(A)** The graphic represents the effect of the Smoking habit Heavy Smokers (HS), Light Smokers (LS), No Smokers (NS) on the frontal alpha asymmetry values reported. **(B)** The graphic plots the interaction PSA kind × Smoking habit. Brackets stand for a statistical significance equal to at least *p* = 0.05, or lower. Error bars represent standard error.

Frontal alpha asymmetry results highlighted a statistically significant effect of the Smoking Habit (*F*_(2,35)_ = 6.229; *p* = 0.005) (Figure 1A), in particular the *post hoc* analysis revealed that lower frontal alpha asymmetry values were reported by NS in comparison to both HS (Cohen’s *d* = 0.746; *p* = 0.003) and LS (Cohen’s *d* = 0.477; *p* = 0.036) groups, while no difference has been observed between HS and LS (Cohen’s *d* = 0.233; *p* = 0.251). Moreover, it has been found a statistically significant interaction between the variables Smoking Habit and PSA kind (*F*_(4,70)_ = 3.226; *p* = 0.017) (Figure 1B). The *post hoc* analysis showed an increase of the frontal alpha asymmetry values relative to the Effective PSAs for the HS group in comparison to both the LS (Cohen’s *d* = 1.898; *p* = 0.002) and NS groups (Cohen’s *d* = 1.536; *p* = 0.004). While there was no statistical difference in the frontal alpha asymmetry values relative to the Ineffective and Awarded PSAs among the experimental groups.

**Figure 2:**
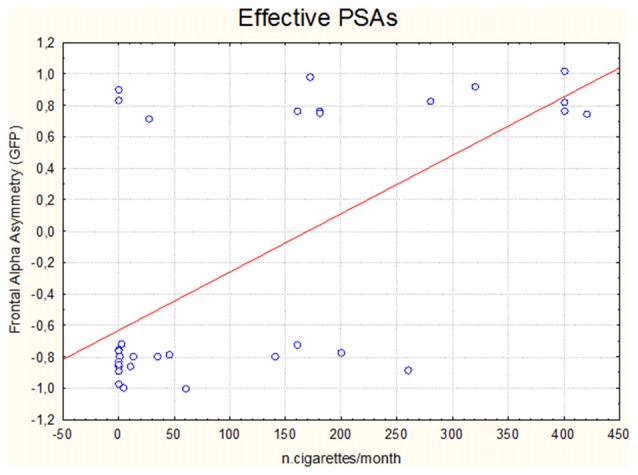
Scatterplot representing the correlation (linear regression analysis) between the number of cigarettes monthly smoked by participants and the relative frontal alpha asymmetry values obtained in correspondence of the exposure to the Effective antismoking public service announcements (PSAs).

In addition, by the linear regression analysis it has been evidenced a correlation between the number of cigarettes regularly smoked by participants and the relative frontal alpha asymmetry values obtained in response to the vision of the Effective PSAs (*η^2^* = 0.423 *R* = 0.650 *p* < 0.001): the higher the number of smoked cigarettes the higher the frontal alpha asymmetry values (Figure 2).

### Frontal Theta

**Figure 3:**
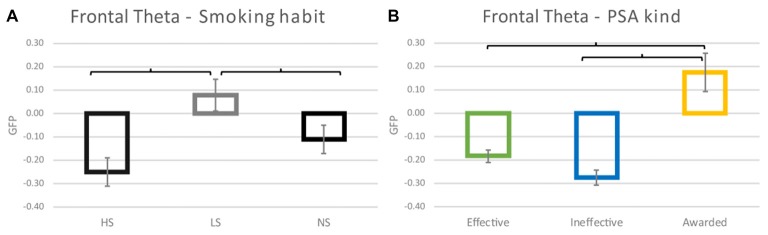
Frontal theta activity results. **(A)** The graphic represents the effect of the Smoking habit (HS, LS, NS) on the frontal theta values reported. **(B)** Plot of the effect of the PSA kind. Brackets stand for a statistical significance equal to at least *p* = 0.05, or lower. Error bars represent standard error.

Frontal theta activity results showed a statistically significant effect of the Smoking Habit (*F*_(2,35)_ = 6.535; *p* = 0.004) (Figure 3A), and the *post hoc* analysis highlited an increase reported by LS in comparison to both HS (Cohen’s *d* = 0.844; *p* = 0.001) and NS (Cohen’s *d* = 0.471; *p* = 0.040), with the HS group reporting the lowest values. Additionally, there was a significant effect of the PSA kind (*F*_(2,70)_ = 24.771; *p* < 0.001) (Figure 3B), characterized by the increase of the values relative to the Awarded PSAs in comparison to the Effective and Ineffective ones (Cohen’s *d* = −0.899 and Cohen’s *d* = −1.098 respectively *p* < 0.001 both).

**Figure 4:**
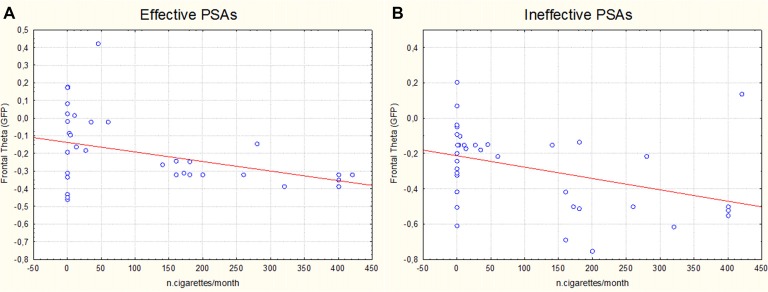
Scatter plots representing the correlation (linear regression analysis) between the number of cigarettes monthly smoked by participants and the relative frontal theta values obtained in correspondence of the exposure to the Effective **(A)** and Ineffective **(B)** antismoking PSAs.

The linear regression analysis revealed a correlation between the number of cigarettes regularly smoked and the decrease of the frontal theta values for both the Effective (*η^2^* = 0.142 *R* = 0.378 *p* = 0.019) (Figure 4A) and Ineffective (*η*^2^ = 0.155 *R* = 0.394 *p* = 0.014) (Figure 4B) PSAs. In other words, the higher the number of cigarettes smoked by participants and the lower the frontal theta values reported.

### Emotional Index

**Figure 5:**
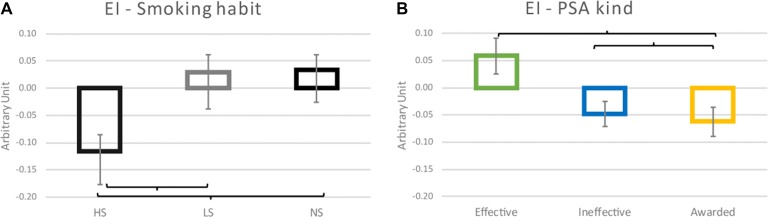
Emotional Index (EI) results. **(A)** The graphic represents the effect of the Smoking habit (HS, LS, NS) on the EI values reported. **(B)** Plot of the effect of the PSA kind. Brackets stand for a statistical significance equal to at least *p* = 0.05, or lower. Error bars represent standard error.

The EI analysis showed a statistically significant effect of the Smoking Habit (*F*_(2,36)_ = 8.139; *p* = 0.001) (Figure 5A), with the HS group expressing a decrease in the EI values in comparison to LS and NS groups (Cohen’s *d* = 0.8 and Cohen’s *d* = 0.852 respectively; *p* = 0.002 both). Furthermore it has been found a statistically significant effect of the PSA kind (*F*_(2,72)_ = 6.470; *p* = 0.003) (Figure 5B), with an increase of the EI values reported for the Effective PSAs in comparison to both the Ineffective (Cohen’s *d* = 0.585; *p* = 0.004) and Awarded (Cohen’s *d* = 0.620; *p* = 0.002) ones.

**Figure 6:**
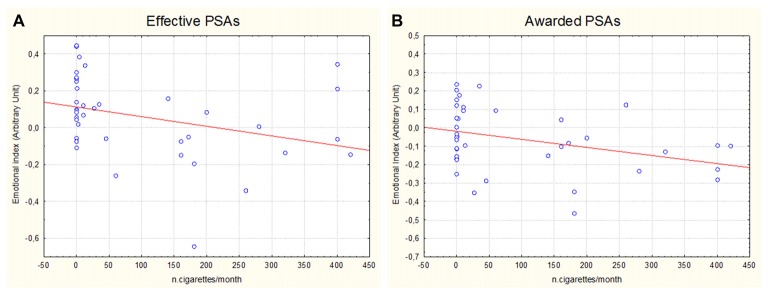
Scatter plots representing the correlation between the number of cigarettes monthly smoked by participants and the relative EI values obtained in correspondence of the exposure to the Effective **(A)** and Awarded **(B)** antismoking PSAs.

There was also a correlation, as evidenced by the linear regression analysis, between the number of cigarettes smoked by participants and the EI values reported in response to the observation of the Effective (*η^2^* = 0.106; *R* = 0.325; *p* = 0.043) (Figure 6A) and Awarded (*η*^2^ = 0.126; *R* = 0.355; *p* = 0.026) (Figure 6B) PSAs: the lower the number of cigarettes smoked and the more positive the corresponding EI values.

### Eye Tracking

**Figure 7:**
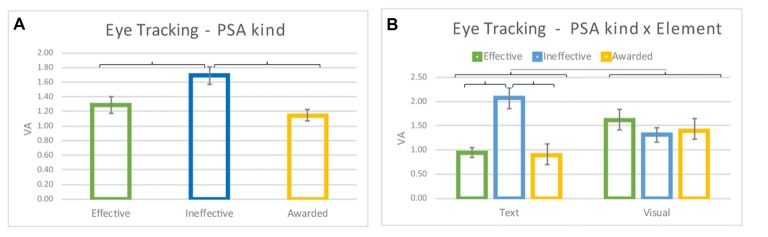
Eye Tracking results. **(A)** The graph represents the effect of the PSA kind (Effective, Ineffective and Awarded). **(B)** The graph represents the interaction between PSA kind and Element (Text and Visual). Brackets stand for a statistical significance equal to at least *p* = 0.05, or lower. Error bars represent standard error.

ET data evidenced a statistically signficant effect of the PSA kind (*F*_(2,50)_ = 8.899; *p* < 0.001) (Figure 7A), with the Ineffective PSA obtaining the higher VA values in comparison to the Effective (Cohen’s *d* = 0.431; *p* = 0.003) and the Awarded (Cohen’s *d* = 0.662; *p* < 0.001) PSAs. Moreover there was a significant interaction between the variable PSA kind and the variable Element (*F*_(2,50)_ = 16.264; *p* < 0.001; Figure 7B). The *post hoc* analysis showed that within each PSA kind there was a significant difference between Text and Visual Element, with the Visual element gaining more VA than the Text element for the Effective (Cohen’s *d* = −0.787; *p* = 0.002) and Awarded (Cohen’s *d* = −0.907; *p* = 0.019), *vice versa* for the Ineffective PSAs the Text element presented higher VA values than the Visual one (Cohen’s *d* = 0.772; *p* = 0.001). In addition, the Text of the Ineffective PSAs obtained higher VA values than the Text elements present in the Effective and Awarded PSAs (Cohen’s *d* = 1.324 and Cohen’s *d* = 1.406 respectively; *p* < 0.001 both), but also than all the Visual elements present in the different kinds of PSAs (Cohen’s *d* = 0.418; Effective *p* = 0.025, Cohen’s *d* = 0.772; Ineffective *p* = 0.001, Cohen’s *d* = 0.770; Awarded *p* = 0.002).

### Behavioral Data

**Figure 8:**
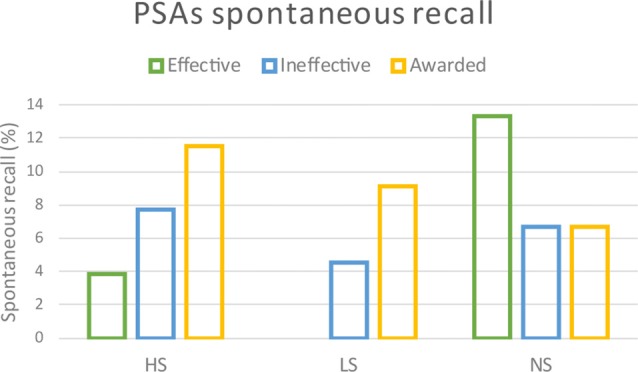
Behavioral results. The graph represents the occurrence of spontaneously recalled Effective, Ineffective and Awarded PSAs by the three experimental groups (HS, LS and NS).

Concerning the spontaneous recall of the PSAs there was a difference in the distribution among the groups (Figure 8). In particular, the NS remembered the Effective PSAs with a higher occurrence in comparison to the HS and LS groups, that instead reported the observation of the Awarded PSAs with a higher percentage. The chi-square test reported a statistical significant difference among the groups (*χ*^2^ > 15.357; *p* = 0.004).

## Discussion

Concerning smoking habits, for the definition of the LS, the threshold of up to five cigarettes per day was selected on the basis of the scientific literature (Evans et al., 1992; Kenford et al., 2005; Brauer et al., 1996; Zhu et al., 2003; Etter, 2004; Owen et al., 1995; Hajek et al., 1995; for a review Schane et al., 2010), although there is not a consensus on the definition of “light smoking” (Husten, 2009). This threshold has been selected since this kind of smoking habit has been found to be particularly spread among young and educated people (Wortley et al., 2003), that was the sample of our study. The identification of this kind of LS appears worthy, since although the daily smoking is declining, the non-daily smoking is increasing (Schane et al., 2009), representing one fourth of the total tobacco users^6^ and being particularly risky since people do not consider themselves as smokers (Fergusson and Horwood, 1995). This despite the possible health-related concerns related to low-exposures, mainly involving cardiovascular disease, lung and gastrointestinal cancers, lower respiratory tract infections, cataracts, compromised reproductive health, and osteoporosis^7^.

### Methodologic Limitations

The present study presents several methodologic limitations that we would like to discuss in this section.

The first issue is related to the sample size investigated for each one of the statistical cells employed. In fact, it may be argued that 12–15 persons per each cell could be not adequate to drawn statistically robust conclusion in a multivariate analysis, especially for the occurrence of the type-I error (Zar, 2000). While the above mentioned limitation is clear, it must be underlined as the ANOVA is a statistical technique that it is robust and it has sufficient statistical power in conjunction with the execution of appropriate *post hoc* tests such as Duncan’s test employed here (Zar, 2000). A second issue is related to the fact that it may be argued as the ANOVAs have been performed without previously checking the normality of the data. While this is true in general, due to the statistical assumptions underlying the ANOVA methodology, it is also true that it has been evidenced how ANOVA is not very sensitive to deviations from normality. In particular, simulation studies using several non-normal distributions have shown that the false positive rate is not affected very much by this violation of the assumption (Glass et al., 1972; Harwell et al., 1992; Lix et al., 1996).

A third issue is related to the material presented for the analysis. It may be argued that since the stimulus materials in this experiment are presented once to each participant, an insufficient number of trials or length for the cerebral and autonomic indexes to be able to detect reliable and valid responses from these neurometric signals could be gathered. However, it has to be noted that we were interested in this study to the reaction of the subjects to the unfamiliar PSAs. In fact, as reported in literature, repeated exposure to a stimulus causes increased liking of that stimulus (Zajonc, 1968). Thus, it makes sense to expose subjects just once to the stimuli to be tested. In addition, recent studies by other research groups follows the adoption of a single exposure to advertising to be tested (Rossiter et al., 2001; Silberstein and Nield, 2012; Yang et al., 2015).

### Frontal Alpha Asymmetry

Hypothesis 2, stated that a higher approach would have been shown by HS in comparison to NS and LS in response to the anti-smoking PSAs. The results here obtained suggested that such hypothesis is true, although, among smokers the statistically significant difference between HS and NS was limited to the Effective PSAs.

The NS group reported the lowest and negative frontal alpha asymmetry values in comparison to HS and LS ones, so suggesting a tendency of avoidance toward the advertised material (Davidson, 2004). Such results are in agreement with the strength of the negative preexisting beliefs about smoking and smokers, in particular after the exposure to antismoking material in youth (Pechmann and Ratneshwar, 1994). On the other hand, HS showed higher frontal alpha asymmetry values in comparison to LS (even if not reaching the statistical significant difference) and the NS. Furthermore, the Effective PSAs included in the present study were characterized by fear arousing appeal and paternalistic communication styles. Those PSAs obtained the more positive frontal alpha asymmetry from HS group in comparison to LS and NS groups. This evidence suggests a tendency of approach that could be symmetrical to the emotional reaction linked to the reactance theory (Brehm and Brehm, 2013). Therefore, the choice of the fear arousing appeal and paternalistic communication styles would be of interest for young smokers, even a possible dangerous trigger of reactance. Furthermore, the negative frontal alpha asymmetry value reported by NS in response to the Effective PSAs suggests that those communication styles (fear arousing appeal and paternalistic) could be effective in young NS in preventing the smoking begin. A similar result has been reported for the LS group in reaction to the vision of Effective PSAs. In this case, those particular PSAs would be effective in preventing the rise of the number of cigarettes daily smoked by participants. These results are basically in accord to the Hypothesis 2, predicting a higher approach tendency by HS in comparison to NS and LS in response to the anti-smoking PSAs. It is indeed interesting that the statistically significant difference between HS and LS was limited to the Effective PSAs. Moreover, the correlation between the number of smoked cigarettes and the frontal alpha asymmetry values in response to the Effective PSAs exposure, could be linked to the quantitative effect of daily tobacco use. In fact, such effect was found to affect the neural response, as measured by the amplitude of the P300 event related potential (Haarer and Polich, 2000). Moreover, the extent of nicotine dependence seems to moderate the cue reactivity toward antismoking stimuli (Vollstädt-Klein et al., 2011). In smokers and non-smokers groups, a different response lateralization between the two hemispheres, in the present study indexed by positive (left) and negative (right) frontal alpha asymmetry values, has been already showed. Specifically, it has been shown an increased P300 amplitude produced by smokers in the right hemisphere in response to both tobacco promoting and antismoking images, *vice versa* in the left hemisphere by non-smokers (Jang et al., 2007). These results appear in contrast with the present data showing a higher response in the left hemisphere in HS and in the right hemisphere in the NS groups, nevertheless this could be attributable to the different definition of smokers between the two researches (i.e., more than two cigarettes per day, while in the present study participants have been divided between HS and LS on the basis of the threshold of five cigarettes per day) and to the different age of the experimental sample (mean age 30.6 years ± 7.5 in Jang et al.’s (2007) study, while 18.308 ± 2.726 years in the present research).

### Frontal Theta

Hypothesis 3 stated that Effective PSAs would have shown increased frontal theta activity in comparison to Ineffective PSAs, mirroring a higher anti-smoking message processing. The results here obtained suggested that such hypothesis is partially true, since in the present sample there was only a trend of such increase. However, it must be noted that repeating the statistical analysis excluding the Awarded PSAs category, while leaving all the other factors unvaried, such increase reached the statistical significance (*F*_(1,35)_ = 5.660, *p* = 0.023).

In fact, the Awarded images, obtained the highest frontal theta levels. This could be explained by the peculiar content and style of the images potentially requiring a high decoding effort, linked to the increase of EEG power in theta band for correctly gaining the antismoking information. In fact, in the case of the picture depicting a cigarette symbolizing an artery filled of fatty deposits, this was probably due to the inferior common knowledge about the vascular consequences of smoking habits among the population^1^ (WHO report 2011), in comparison to the more famous pulmonary effects. Moreover, in the case of the young man smoking a cigarette, it could be explained by the sarcastic nature of the sentence above and below him. Furthermore, present data agrees with evidences of a discrimination of awarded advertising through, among the others, EEG theta band, in the right fronto-temporal regions (Wang et al., 2018). In addition, it has been evidenced that in moderately dependent smokers brain activity elicited by tobacco advertisements was higher in comparison to highly dependent smokers (Vollstädt-Klein et al., 2011). This phenomenon could also take shape in the higher frontal theta levels identified in the LS group in comparison to the HS and NS groups. This result suggests that LS group would be particularly receptive to aesthetic features in anti-smoking PSAs, thus producing a spontaneous targeting of the PSA that could be employed for the delivery of the messages to that specific population.

Finally, the correlation between the number of cigarettes regularly smoked by participants and the EEG frontal power in theta band values suggests that HS would percept Effective antismoking PSAs as less effortful or eliciting a lower processing than the NS group. This could be connected to the reason why those participants actually did not smoke, that is, NS participants performed a higher cognitive processing of the antismoking content of the PSAs in comparison to HS group. The same consideration could be extended also to the correlation between the number of smoked cigarettes and the EEG frontal power in theta band in correspondence of the exposure to Ineffective PSAs, even if in this case the cerebral frontal theta resulted to be unuseful for the obtainment of compelling antismoking messages. About the levels of frontal theta elicited by the Effective PSAs, the pre-test of the advertising for the frontal theta evaluation in target populations appears useful. Concerning the Awarded PSAs, the absence of a statistical correlation with the number of cigarettes smoked by participants support the hypothesis formulated above of their stylistic complexity in general, not promoting attitudinal/beliefs changes in the population, so without differences among the groups.

### Emotional Index

Hypothesis 1, according to the theory of the psychological reactance, stated that HS would present a more negative emotional reaction to antismoking PSAs in comparison to LS and NS. The results here obtained suggested that such hypothesis is true.

Concerning EI results, the evidence that HS group presented the most negative emotional reaction toward stimuli can be linked to the theory of psychological reactance (Brehm and Brehm, 2013), that states why efforts to convince adolescents not to smoke may lead to an opposite effect. In addition, it has been shown that youth smoking prevention advertisements have been rated more negatively by adolescents with high psychological reactance than participants with low psychological reactance (Henriksen et al., 2006). Therefore, it is possible to hypothesize that HS participants presented a higher potential for reactance, and an indicator of the oppositional attitudes of adolescents toward authority are associated with tobacco use (Wakefield et al., 2005). It could be hypothesized that the cognitive reaction, as estimated by the frontal alpha asymmetry index, toward the Effective PSAs is symmetrical to the emotional reaction emboding the reactance response. The sum of these evidences suggests that in young HS group the Effective PSAs would elicit positive frontal alpha asymmetry values and negative EI values. Therefore, this pattern of indicators levels could be the best mix to realize antismoking PSAs able to promote quitting. In fact, the Effective PSAs are campaigns that already received a favorable acceptance by the public. It is indeed reasonable that the PSA material from the governs could be usefully pre-tested in such population in order to estimate the neurometric reaction before their launch on air. The verification of the presence of such pattern in frontal alpha asymmetry and negative EI values before the launch could reinforce the idea that the advertising could be a success, and viceversa.

The correlation between the number of cigarettes regularly smoked by participants and the EI values reported in response to the Effective and Awarded PSAs suggests that congruent, threatening and well-delivered antismoking messages would elicit a greater emotional reaction in smokers than in non-smokers, probably because as felt as more involving themselves.

Looking at the two extremes of the smoking habit behavior (HS and NS), summing evidences from frontal alpha asymmetry, frontal theta and EI, Effective PSAs should present in *a priori* test different patterns. In particular, PSAs to be effective in HS would present positive (approach tendency) frontal alpha asymmetry, low frontal theta and negative EI levels; while in NS Effective PSAs would present negative (withdrawal tendency) frontal alpha asymmetry, higher frontal theta and more positive EI values. These two patterns would be promoting two distinct reactions: “quitting” in HS and “prevention from starting” in NS, respectively.

### Eye Tracker

Hypothesis 3 stated that *a priori* assessment of effective anti-smoking material is reliable. The results here obtained suggested that such hypothesis is true.

The analysis of the VA index showed that participants tended to look more at the informative elements (text or graphical elements) of the Ineffective PSAs. This result could be explained by the poor delivery of the message promoted by this kind of PSAs in accordance with a study by Rayner (2009), where is highlighted a direct correlation between the time spent on an AOI and the inability of comprehension of the message: smoking cessation habits in this particular case. Looking at the interaction of PSAs kind and the Elements (Text and Visual) of such PSAs, it is evident the presence of different trends in the VA for the different PSAs categories. In particular, in the Effective and in the Awarded PSAs the visual elements were the most attractive in contrast to the Ineffective PSAs, where the texts seemed to be the main focus of attention. This could reflect the lack of information about anti-smoking contents in the Ineffective PSAs, where the perceived message appears unclear and poorly related to the issue. Specifically in fact, the Ineffective PSAs were constituted by: one visual element consisting in a set of young models without any reference to the tobacco consumption (Feel free to say no PSA), and the second was a drawing of a coughing boy, but in a cluttered and hard to decode picture, where the embedded antismoking message was probably difficult to perceive (Pieters et al., 2007).

Evidences suggest that the text is the main focus on a printed advertising (Rayner et al., 2001), but in the present study concerning antismoking campaigns, this holds true only for the Ineffective PSAs.

This could be explained by the reason that for the Effective and Awarded PSAs, where the message proposed by the visual element was clearer and more immediate (e.g., the lady who underwent the tracheotomy), there was less needing to obtain information from the textual element. The graphical elements in this case would be self-explanatory and strictly related to the issue, so there was no need to further investigate the purpose or the message of the campaign by spending more time on the text elements.

The results from ET strongly suggest that Effective PSAs should present a clear and immediate message, strictly connected to the textual element. Therefore *a priori* pre-test of the PSA would be useful for the evaluation through VA metrics of the ET pattern elicited by the advertisement.

## Conclusion

In conclusion, we could state that the present study supports almost all the hypothesis for the evaluation of antismoking PSA with the neurophysiologic measurements. Specifically, of the three Hypothesis at the base of the study, the Hypothesis 1 and 3 was fully supported by the experimental data. Hypothesis 2 has been found instead supported by the experimental data only for the part relative to the Effective PSAs.

## Author Contributions

GC and DR performed the statistical analysis and wrote the manuscript. EM and DR performed the data analysis. GC, EM, DR, PC, MM and AM performed the data recording. FB, AT and AC supervised the project and edited the manuscript.

## Conflict of Interest Statement

The authors declare that the research was conducted in the absence of any commercial or financial relationships that could be construed as a potential conflict of interest.

## Footnotes

^1^http://www.who.int/tobacco/global_report/2011/en/

^2^http://www.who.int/tobacco/global_report/2013/en/

^3^The role of the media in promoting and reducing tobacco use. Bethesda, MD, US Department of Health and Human Services, National Institutes of Health, National Cancer Institute, 2008 (Tobacco Control Monograph No. 19, NIH Pub. No. 07-6242). Available online at: http://www.cancercontrol.cancer.gov/brp/tcrb/monographs/19/index.html

^4^Social determinants of health and well-being among young people. Health behavior in school-aged children (HBSC) study: International report from the 2009–2010 survey. Geneva, World Health organization, 2012. Available online at: http://www.euro.who.int/__data/assets/pdf_file/0003/163857/Social-determinants-of-health-and-well-being-among-young-people.pdf

^5^Il fumo in Italia—ISS-DOXA 2015. Available online at: http://www.iss.it/fumo/index.php?lang=1&id=350&tipo=18

^6^Centers for Disease Control and Prevention (CDC), Prevalence of current cigarette smoking among adults and changes in prevalence of current and some day smoking—United States, 1996-2001. Morb. Mortal Wkly. Rep. 2003;52 (14) 303–307

^7^Office of the Surgeon General (US), & Office on Smoking and Health (US). (2004). The Health Consequences of Smoking: A Report of the Surgeon General. Atlanta (GA, USA): Centers for Disease Control and Prevention (US). Available online at: http://www.ncbi.nlm.nih.gov/books/NBK44695/
